# Necessity of Caution in the Employment of Chloroform during Labor

**Published:** 1877-08-20

**Authors:** W. T. Lusk

**Affiliations:** New York


### NECESSITY OF CAUTION IN THE EMPLOYMENT OF CHLOROFORM DURING LABOR.

Dr. W. T. Lusk, of New York, read a paper upon the above subject at the American Gynecological Society, and presented the following propositions:

1. Deep anæsthesia, carried to the point of complete abolition of conscious ness, retarded, sometimes suspended uterine action. It was that fact which made it so valuable in many cases, but safety required that the woman should come partially from under its influence before completing labor, in order to avoid hemorrhage.
2. Chloroform, when given in the usual obstetric fashion, in exceptional cases, so far weakens uterine action as to create a necessity for resorting to ergot and the forceps.
3. Women in labor did not enjoy any absolute immunity from the deleterious effects of chloroform. A number of cases were related, and the Doctor was not willing to believe they were exceptional.
4. Chloroform should not be given in the *third* stage of labor.

5. The more remote influence of large doses of chloroform during labor upon the puerperal state was a subject which called for farther investigation and inquiry.

Dr. Wilson, of Baltimore, remarked that the cases referred to by Dr. Lusk were the first he had heard reported of death from chloroform occurring during parturition. He had used chloroform between two and three thousand times, and with not a single bad result. He always preceded its inhalation by the administration of a dose of some alcoholic stimulant.

Dr. A. H. Smith of Philadelphia, employed Squibb's ether in ordinary obstetric practice, because it possessed all the advantages of chloroform and was absolutely safe. There were cases in which he wished to produce absolute relaxation and loss of consciousness, and rapidly—for example, for the purpose of instantly carrying the finger to the fundus and removing an ovum, in cases of hemorrhagic abortions, then chloroform was the better agent, and for the short time the patient was under its profound influence, it was ordinarily safe.—*Med. Times.*

